# Pneumocystis pneumonia secondary to intensive immunosuppression treatment for anti-GBM disease complicated with IgA nephropathy: A case report and literature review

**DOI:** 10.1097/MD.0000000000027728

**Published:** 2021-11-12

**Authors:** Manyu Zhang, Dingwei Yang, Weixiu Wang, Fuhao Zhao, Xiaoxiao Zhang, Xue Li

**Affiliations:** a Department of Nephrology, Tianjin Hospital, Tianjin, China; b Tianjin Medical University, Tianjin, China.

**Keywords:** anti-GBM disease, case report, IgA nephropathy, immunosuppression treatment, pneumocystis pneumonia

## Abstract

**Rationale:**

The estimated incidence of anti-glomerular basement membrane (anti-GBM) disease complicated with immunoglobulin A (IgA) nephropathy is minimal, there have only been 15 cases (including this case) reported in the literature, and only 5 (33.33%) of them showed significant improvement in renal function after treatment. Pneumocystis pneumonia is a severe opportunistic pulmonary infection of pneumocystis jiroveci in immunocompromised patients. Here, we report a case of pneumocystis pneumonia secondary to intensive immunosuppression treatment for anti-GBM disease complicated with IgA nephropathy, with no similar reports or studies published before to our knowledge.

**Patient concerns:**

The patient was admitted to our hospital with a 1-week diagnosis of crescent glomerulonephritis who had been suffered from hematuria and foamy urine for more than 1 month. Before admission, the patient received pulse dose intravenous methylprednisolone and immunosuppression with rituximab, but the renal function and titer of pathogenic antibody did not improve significantly.

**Diagnosis:**

Crescentic glomerulonephritis, anti-glomerular basal membrane disease complicated with IgA nephropathy (Type I+II) was pathologically confirmed by renal biopsy. Secondary pneumocystis pneumonia was diagnosed by acute progressive respiratory failure, chest computed tomography and metagenomic next-generation sequencing of transbronchoscopic bronchoalveolar lavage fluid.

**Interventions:**

The key to successful treatment was to make the pathogenic antibody turn negative quickly by combining pulse dose intravenous methylprednisolone, immunosuppression with rituximab, and plasma exchange therapy. Early identification of pneumocystis pneumonia, accurate etiological identification, and active anti-infective treatment were also crucial.

**Outcomes:**

The patient was discharged after 16 days of anti-infection with secondary infection controlled and dialysis catheter removed. Up to now, the patient has been followed for a period of 28 weeks, results showed renal function had been repaired even hematuria and proteinuria were basically alleviated.

**Lessons:**

Our case provided experience in the treatment of anti-GBM disease complicated with IgA nephropathy, further proposed the potential therapeutic effects of rituximab, also illustrated low dose hormone combined with tacrolimus can be used as sequential therapy after plasma exchange and intensive immunosuppression. Our research also suggested that resulting in severe immune suppression, a high risk of secondary pneumocystis opportunistic infection should be aware of. metagenomic next-generation sequencing might increase the detection rate of the pathogen.

## 1 Introduction

Anti-glomerular basement membrane (anti-GBM) disease classically presents with aggressive necrotizing and crescentic glomerulonephritis, often with pulmonary hemorrhage. Classic cases are diagnosed based on the presence of the anti-GBM antibody in serum samples and kidney or lung biopsy tissue samples,^[1]^ the estimated incidence of anti-GBM disease is <2 cases per million population per annum, it is even lower in Asian populations.^[2,3]^ The primary therapeutic aim is to render the anti-GBM antibody titer negative as quickly as possible combining plasmapheresis and immunosuppression.^[4]^ Plasmapheresis is recommended in all patients with anti-GBM disease except those who are dialysis dependent at presentation, have 100% glomerular crescents, and do not have pulmonary hemorrhage.^[5,6]^ In the concomitant diseases of anti-GBM disease, antineutrophil cytoplasmic antibody (ANCA) associated vasculitis has the highest incidence of 30% to 40%^[3]^. In the contrary, there have only been 15 case reports (including this case) of anti-GBM disease complicated with IgA nephropathy which were reported in the literature,^[7–20]^ only 5^[10,12,16,17]^ (33.33%) of them showed significant improvement in renal function after treatment, treatment experience is obviously insufficient.

Severe infections were frequent during the early phase of the disease and were associated with substantial morbidity and a reduced 3-year survival rate. The lungs, the urinary tract, and catheters were the main sites of infection.^[21]^ Pneumocystis pneumonia (PCP) is a severe opportunistic pulmonary infection of pneumocystis jiroveci in immunocompromised patients, which mortality is as high as 29% to 50%.^[22,23]^ Patients with kidney disease who receive immunosuppressive therapy are among the high-risk groups. Metagenomics second-generation sequencing (mNGS) technology has been gradually applied to the diagnosis of infectious diseases in recent years.^[24]^ Although its clinical practice data still need to be further accumulated, the technology has shown a broad application prospect.

## 2 Case presentation

**Figure 1:**
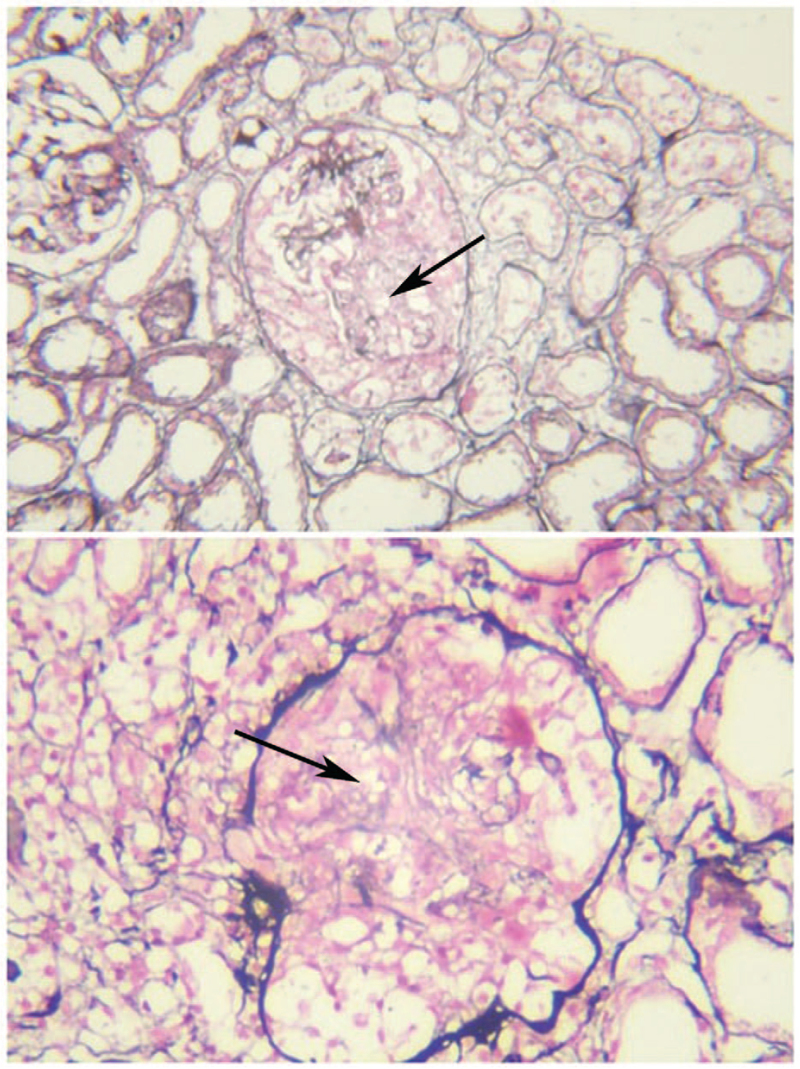
Pathological changes. Light microscopy (PASM+Masson, the amplification times: 400) of renal puncture tissue revealed the formation of large crescents, the pathological manifestations were dominated by cellular crescents, and there was a tendency toward cellular-fibrous crescents. Immunofluorescence failed to preserve images due to equipment reasons.

A 41-year-old female was admitted to the hospital with a 1-week diagnosis of crescent glomerulonephritis who had been suffered from hematuria and foamy urine for more than 1 month. Half a month before admission, the patient developed fever, cough, and expectoration, with the highest body temperature of 38.5°C. Then, the patient was tested for serum creatinine 157.6 μmol/L (the normal reference range: 57–111 μmol/L) in another hospital, and was given piperacillin-tazobactam for anti-infection, supplemented with treatment for kidney preservation and circulation improvement. However, posttreatment review revealed a gradual increase in creatinine, Urinary sediment showed 476.69 red blood cells/high power field (HPF) (the normal reference range: 0–2.4/HPF), and urinary protein excretion was 2.137 g/24 h (the normal reference range: 28–141 mg/24 h). The patient's serum anti-GBM antibody titer was markedly elevated to 47.23 RU/mL (the normal reference range: <20 RU/mL), with negative results for serum antinuclear antibody and ANCA. With the guidance of ultrasonography, renal biopsy was carried out to ascertain the cause. After the renal biopsy, methylprednisolone (40 mg intravenous drip, once a day) was added for treatment, then the body temperature was controlled, the cough and expectoration was also significantly reduced, but the serum creatinine rose rapidly to 278.3 μmol/L (the normal reference range: 57–111 μmol/L). According to the diagnosis of crescent nephritis based on the urgent pathological examination, the patient was treated with pulse dose intravenous methylprednisolone 1000 mg/d for 3 days followed by maintenance intravenous methylprednisolone at 40 mg/d. Besides, on the third day of pulse dose intravenous methylprednisolone therapy, Rituximab (500 mg intravenous drip) was given in combination to enhance the immunosuppressive effect. Based on complete pathologic findings, the patient was diagnosed as crescentic glomerulonephritis (Fig. 1), antiglomerular basal membrane disease complicated with IgA nephropathy (Type I+II). After 2 days of pulse dose intravenous methylprednisolone therapy, the patient developed fatigue, abdominal distension, nausea, intermittent vomiting, and edema in both lower extremities. Four days later, the serum creatinine continued to increase to 402.6 μmol/L (the normal reference range: 57–111 μmol/L) with the serum anti-GBM antibody rose to 100.8 RU/mL (the normal reference range: <20 RU/mL). Considering the continuous progress of the patient's renal function and the increasing trend of anti-GBM antibody, the patient was transferred to our department for further treatment.

The patient was healthy in the past with no relevant medical history. On the day of admission, blood and urine samples were collected for examination, and the results revealed serum creatinine level of 374 μmol/L (the normal reference range: 57–111 μmol/L), serum anti-GBM antibody titer of 77.107 RU/mL (the normal reference range: <20 RU/mL), urinary occult blood count of 3+(797cells/HPF with the normal reference range: 0–2.4/HPF) and 24 h urinary protein of 2452.5 mg/24 h (the normal reference range: 28–141 mg/24 h). Other parameters are shown in Table 1.

**Table 1:** Routine lab results on admission (The normal reference ranges are presented in footnotes^∗^.).

White blood cell (WBC, × 10^9^/L)	16.54	Neutrophil (NEU%, %)	81.50	Immunoglobulin G (IgG, mg/dL)	619
Hemoglobin (HGB, g/L)	91	Platelet (PLT, × 10^9^/L)	394	Complement 3 (C3, mg/dL)	77.9
Urea (mmol/L)	14.6	C-protein (CRP, mg/L)	31.1	Anti-nuclear antibody (ANA)	−
Uric acid (UA, μmol/L)	436	Erythrocyte sedimentation rate (ESR, mm/h)	115	Antineutrophil cytoplasmic antibody (ANCA)	−
Albumin (ALB, g/L)	33.7				

∗ WBC: 3.5–9.5 × 10^9^/L, NEU%: 40–75%, IgG: 751–1560 mg/dL, HGB: 130–175 g/L, PLT: 125–350 × 10^9^/L, C3: 79–152 mg/dL, Urea: 3.1–8.0mmol/L, CRP: 0–8 mg/L, ANA: negative(−), UA: 208–428 μmol/L, ESR: 0–15 mm/h, ANCA: negative(−), ALB: 40–55 g/L.

Based on these auxiliary examination results and disease evolution process, the patient was diagnosed as acute renal failure, crescentic glomerulonephritis (Crescentic glomerulonephritis are characterized by a crescent-shaped cellular proliferation that may lead to glomerular destruction. Over 50% of at least 10 analyzed glomeruli should be affected, and the crescents always account for more than 50% of renal vesicles.), antiglomerular basal membrane disease complicated with IgA nephropathy (Type I+II). Accordingly, the patient received plasma exchange (PE) for 6 sessions, hemodialysis was performed 3 times according to urine volume and creatinine level. She was treated with maintenance intravenous methylprednisolone at 40 mg/d and intravenous immunoglobulin therapy for 12 consecutive days. As a result, her serum anti-GBM antibody titers turned negative on Day 12.

**Figure 2:**
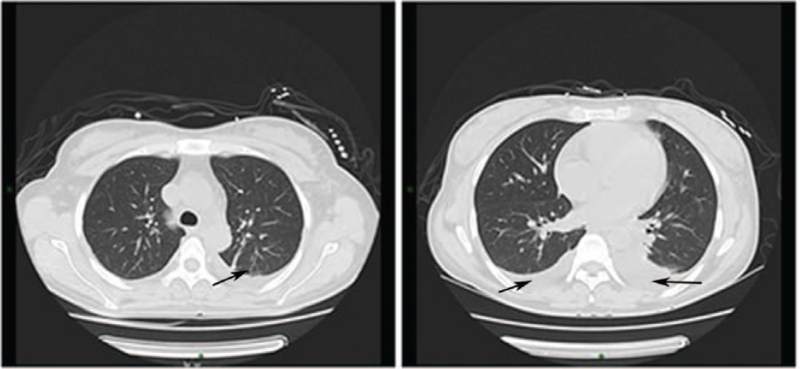
Chest CT on Day 8. Bilateral pleural effusion and local atelectasis; ground glass density shadow and strip shadow in adjacent lung field were considered to be associated with chronic inflammation.

On the 8th day of treatment, the patient developed intermittent dry cough, no phlegm, no fever, and no abnormality on auscultation. Considering that the patient has used pulse dose intravenous methylprednisolone therapy and was still receiving sequential treatment with glucocorticoids and plasma exchange, secondary infection was highly suspected. Chest CT examination was performed, which showed bilateral pleural effusion, local atelectasis, and chronic inflammation (Fig. 2), moxifloxacin and cefoperazone/sulbactam were used for anti-infection treatment.

On the 10th day, the patient developed dry cough again with no phlegm, no fever, stable vital signs, and no abnormality on auscultation. Routine antibiotics were given on the day of infusion. The patient received plasma exchange treatment on Day 11, she returned to the ward with a headache and a slight increase in body temperature complaining “got cold in the process of transport and treatment.” After taking “compound acetaminophen,” the headache relieved.

**Figure 3:**
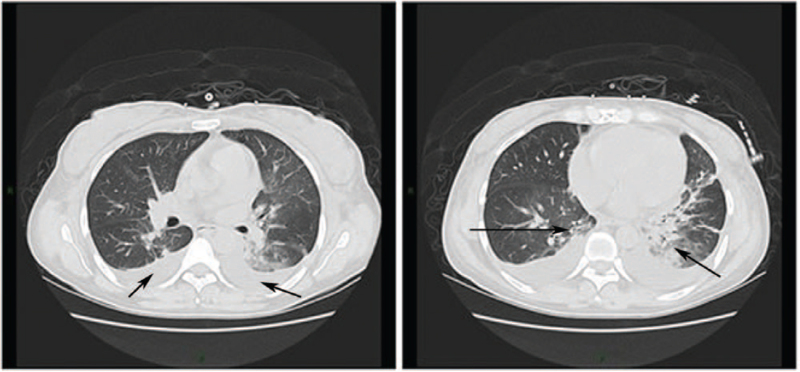
Chest CT on Day 12. Bilateral ground-glass opacity, atelectasis in the lower lobes of both lungs, especially in the left lung. Bilateral pleural effusion and a small amount of interlobar effusion on the left side.

On the 12th day, the patient appeared mentally weak, with intermittent cough, no phlegm, and no fever. Percutaneous oxygen saturation decreased to 83% to 88% (the normal reference range: 95%–100%) under 21% of fraction of inspired oxygen (FiO_2_), and scatter rales in both lower lungs on auscultation. Arterial blood gas indicated respiratory failure (pH 7.404 [the normal reference range: 7.35–7.45], partial pressure of oxygen 55.4 mmHg (the normal reference range: 80–100 mmHg), partial pressure of carbon dioxide 37.9 mmHg (the normal reference range: 35–45 mmHg), HCO_3_- 23.6 mmol/L (the normal reference range: 21–28 mmol/L), oxygen saturation 84.4% (the normal reference range:92%–99%), oxygen partial pressure difference of alveoli-artery 53.9 mmHg (the normal reference range: 0–20 mmHg, FiO_2_ 21.0%). The re-examination of chest CT appeared bilateral ground-glass opacity indicated lung infection (Fig. 3).

The patient developed to respiratory failure within 1 day, in combination with chest CT findings and the rapid progression of the disease, for the sake of the patient's high possibility of opportunistic infection, the PCP was considered. The patient continued to have no phlegm, so transbronchoscopic bronchoalveolar lavage fluid was carried for high-throughput gene detection of pathogenic microorganisms. Before the results were repaid, we have already administered meropenem, linezolid, compound sulfamethoxazole, and caspofungin as a combination of potent anti-infective therapy actively. After 1 day of quadruples anti-infective treatment, the general state of the patient was significantly improved, self-reported cough was relieved, percutaneous oxygen saturation was 93% to 95% (the normal reference range: 95%–100%) under 21% of FiO_2_, oxygen inhaled by nasal catheter for 4 L/min could be increased to 98% to 99% (the normal reference range: 95%–100%), and re-examination of arterial blood gas was significantly improved (pH 7.360 [the normal reference range: 7.35–7.45], partial pressure of oxygen 71.8 mmHg [the normal reference range: 80–100 mmHg], partial pressure of carbon dioxide 39.2 mmHg [the normal reference range: 35–45 mmHg], HCO_3_- 21.9 mmol/L [the normal reference range: 21–28 mmol/L], oxygen saturation 94.9% [the normal reference range:92%–99%], oxygen partial pressure difference of alveoli-artery 123.0 mmHg [the normal reference range: 0–20 mmHg], FiO_2_ 33.0%). mNGS of transbronchoscopic bronchoalveolar lavage fluid indicated pneumocystis jevi (4446 copies) that night. Accordingly, the current anti-infection regimen was maintained, plasma exchange was postponed, and methylprednisolone was reduced to 30 mg/d. Changes in blood routine, urine routine, renal function, and serum anti-GBM antibody titer were dynamically monitored (Table 2). The treatment of linezolid was cut off 4 days later. After 16 days of continuous application of the rest 3 drug, compound sulfamethoxazole continued to be used after discharge to prevent infection. The chest CT was rechecked 3 weeks after discharge, and the infection was completely absorbed without any obvious abnormalities.

**Table 2:** Dynamic changes of blood routine, urine routine, renal function, and anti-GBM antibody titer after the treatment (The normal reference ranges are presented in footnotes^∗^.).

	D1	D3	D12	D15	D17	D20	D25
CREA^†^(μmol/L)	374	337	395	427	417	347	312
Anti-GBM^‡^(RU/mL)	77.107	36.104	18.169	/	/	18.679	/
WBC^§^(×10^9^)	16.54	23.85	21.26	8.49	9.54	10.95	9.83
NEU^||^(%)	81.5	87.5	81.9	76.3	74.6	68.8	64.0
CRP^¶^(mg/L)	31	13	5	9	/	/	5
ESR^∗∗^ (mm/h)	115	/	54	/	/	60	/
24 h-PRO^††^(mg/24 h)	2452.5	/	5913.6	/	/	4573.5	/
Urinary RBC^‡‡^ (cells/HPF^§§^^)^	797 (80%)	405 (40%)	549 (30%)	/	664 (30%)	/	/

∗ CREA-S: 57–111 μmol/L, Anti-GBM<20 RU/mL, WBC: 3.5–9.5 × 10^9^/L, NEU%: 40%–75%, CRP: 0–8 mg/L, ESR: 0–15 mm/h, 24 h PRO: 28–141 mg/24 h, Urinary RBC: 0–2.4 cells/HPF.

† CREA = creatinine.

‡ GBM = anti-glomerular basement membrane.

§ WBC = white blood cell.

|| NEU = neutrophil.

¶ CRP = C-protein.

∗∗ ESR = erythrocyte sedimentation rate.

†† 24 h-PRO = 24 h-urinary protein.

‡‡ RBC = red blood cell.

§§ HPF = high power field.

After treatment, the general state of the patient was significantly improved, with no fever, no cough and sputum, no chest tightness, no nausea, and vomiting. The edema subsided and the urine volume was maintained at 2000 to 2500 mL/d. With the infection under control, the patient's serum creatinine dropped, the dialysis catheter was removed, and the serum creatinine was maintained at 300 to 350 μmol/L (the normal reference range: 57–111 μmol/L). After discharge, triamcinolone combined with tacrolimus was taken orally regularly to suppress immunity, renal function was gradually improved. As the anti-GBM antibody continued to be negative, tacrolimus was discontinued 16 weeks after discharge, and the dose of triamcinolone was gradually reduced. Up to now, the patient has been followed for a period of 28 weeks, the maintenance dose of triamcinolone has been reduced to 4 mg/d, the serum anti-GBM antibody was persistently negative, the patient was dialysis independence with serum creatinine fluctuated from a low of 117.5 μmol/L (the normal reference range: 57–111 μmol/L) to a high of 191 μmol/L (the normal reference range: 57–111 μmol/L), all follow-up parameters were shown in Table 3.

**Table 3:** Follow-up parameters after discharge (The normal reference ranges are presented in footnotes^∗^).

Date (after discharge)	Anti-GBM^†^ (RU/mL)	urinary RBC^‡^ (cells/μL)	PRO^§^ (g/24 h)	ALB^||^ (g/L)	Crea^¶^ (μmol/L)	WBC^∗∗^(×10^9^)	NEU^††^ (%)	HGB^‡‡^ (g/L)	PLT^§§^, (×10^9^/L)
1 wk	12.122	505		44.5	279	15.76	82.80	138	364
3 wks		700 (60%)	2.17	39.3	200	21.16	85.50	124	233
5 wks		1237 (60%)				14.47	81.10	113	197
6 wks	3.553	653	1.663	38.7	191				
7 wks	2.329	573	1.134	39.3	178	12.65	79.30	111	197
9 wks		423		39.7	153	9.06	73.60	107	182
11 wks		323	0.934		156	14.10	76.80	112	189
14 wks		434	0.362	42.7	166	22.97	70.20	103	201
18 wks	2			36.9	135	7.76	90.10	96	193
22 wks		65	−	39.1	117.5	13.51	72.50	94	246
28 wks	−	53	−		151.7				

∗ Anti-GBM: negative/<20RU/mL, urinary RBC: 0–17 cells/μL, PRO: negative/28–141 mg/24h, ALB: 40–55 g/L, CREA: 57–111 μmol/L, WBC: 3.5–9.5 × 10^9^/L, NEU%: 40%–75%, HGB: 130–175 g/L, PLT: 125–350 × 10^9^/L.

† GBM = anti-glomerular basement membrane.

‡ RBC = red blood cell.

§ PRO = 24 h-urinary protein.

|| ALB = albumin.

¶ CREA = creatinine.

∗∗ WBC = white blood cell.

†† NEU = neutrophil.

‡‡ HGB = hemoglobin.

§§ PLT = platelet.

## 3 Discussion/Conclusion

Anti-GBM disease is an organ-specific autoimmune disease that involves the lung and kidneys and leads to rapid glomerulonephritis progression, with or without diffuse alveolar hemorrhage, and even respiratory failure. Classic cases of anti-GBM disease are diagnosed based on the presence of the anti-GBM antibody in serum samples and kidney or lung biopsy tissue samples,^[1]^ the estimated incidence of anti-GBM disease is <2 cases per million population per annum, it is even lower in Asian populations.^[2,3]^ In this case, the patient was admitted with clinical manifestations of rapidly progressive glomerulonephritis. The significantly abnormal high concentration of anti-GBM antibody in serum samples and percutaneous renal biopsy both supported the diagnosis of crescent glomerulonephritis and anti-glomerular basal membrane disease. In particular, the patient was accompanied by immunoglobulin A (IgA) granular mesangial deposition and hyperplastic changes in mesangial cells and mesangial matrix, all the above supported the diagnosis of anti-GBM disease complicated with IgA nephropathy. ANCA vasculitis has the highest incidence of concomitant disease with anti-glomerular basement membrane disease, up to 30% to 40%^[3]^. Most of the ANCA/anti-GBM double-positive patients initially develop ANCA-related vasculitis. The tissue injury from ANCA vasculitis probably exposes the usually sequestered antigenic epitopes triggering the development of anti-GBM antibody leading to glomerular and/or pulmonary damage.^[25]^ Patients with coexisting anti-GBM disease and membranous nephropathy have also been reported, with mild renal impairment but more prominent proteinuria.^[26,27]^

Through searching domestic and foreign databases, there have been 15 (including this case) case reports of anti-GBM disease complicated with IgA nephropathy.^[7–20]^ (Table 4) Some authors believe that such patients may have pre-existing IgA nephropathy, the release of inflammatory mediators in renal tissue leads to the conformational change of glomerular basal membrane protein, and the increase of antigen exposure promotes the production of antibodies. Second, abnormalities of IgA molecules in IgA nephropathy might be another factor in the pathogenesis of anti-GBM disease with IgA nephropathy, the deposition of aberrant IgA1 along GBM might induce novel antigen formation and consequently lead to the production of anti-GBM antibody,^[8]^ however, the exact mechanism is still not clear.

**Table 4:** Published cases of anti-GBM^∗^ disease complicated with IgA^†^ nephropathy (the normal reference range are presented in footnotes^‡^).

Case	Creatinine on admission (μmol/L)	Erythrocyturia	Proteinuria	Anti-GBM antibody	Treatment	Outcome
1^[7]^	758.3	Full field	9.25 g/24 h	+	Blood purification/ mycophenolate Mofetil (MMF)	Loss to follow-up
2^[8]^	1347	/	/	+, 134%	/	Maintenance dialysis (MHD)
						
3^[9]^	287	4+	3.76 g/24 h	93.5 RU/mL	Pulse dose of intravenous methylprednisolone/PE^§^/sequential steroid combined with cyclophosphamide	Anti-GBM antibody turned negative after 29 ds, creatinine was 375 μmol/ L.
4^[10]^	505	/	2.2 g/24 h	237.6 RU/mL	Pulse dose of intravenous methylprednisolone/sequential steroid combined with cyclophosphamide	Anti-GBM antibody rechecked negative after 4 months, creatinine was 196 μmol/ L.
5^[11]^	884	5061.6 cells/μL	3+	+	Pulse dose of intravenous methylprednisolone and cyclophosphamide/double filtration plasmapheresis/intravenous immunoglobulin (IVIG)/blood purification	MHD^||^
6^[12]^	232	10–15 cells/HPF^¶^	0.41 g/24 h	258.3 RU/mL	Pulse dose of intravenous methylprednisolone/sequential steroid combined with MMF^∗∗^	Creatinine rechecked was 74 μmol/L after 2 yrs.
7^[13]^	373.9	941.9 cells/HPF	2+	3+	Pulse dose of intravenous methylprednisolone/PE/sequential steroid combined with cyclophosphamide	MHD
8^[14]^	247.9	3+	3+	negative 19.7 RU/mL	Pulse dose of intravenous methylprednisolone and cyclophosphamide/PE/IVIG^††^/sequential steroid monotherapy	Loss to follow-up
9^[15]^	282.88	3+	3+	96 RU/mL	Pulse dose of intravenous methylprednisolone /sequential steroid monotherapy	Non-dialysis-dependent chronic renal failure
10^[16]^	411	211 cells/μL	2.2 g/24 h	141 RU/mL	Pulse dose of intravenous methylprednisolone and cyclophosphamide/double filtration plasmapheresis/IVIG/sequential steroid monotherapy	Creatinine rechecked was 133 μmol/L after 1 mo.
11^[17]^	481.78	/		187.2 RU/mL	Pulse dose of intravenous methylprednisolone and cyclophosphamide	Anti-GBM antibody turned negative after 3 months, creatinine was 183.88 μmol/ L.
						
12^[18]^	77	250 cells/μL	0.5 g/24 h	+	PE/sequential steroid combined with cyclophosphamide/sequential steroid combined with azathioprine 2 mo later	The patient was admitted with respiratory symptoms and renal condition was basically stable.
13^[19]^	1387.88	25–30 /HPF	7.04 g/24 h	+	Pulse dose of intravenous methylprednisolone/ sequential steroid combined with cyclophosphamide/ blood purification	MHD
						
14^[20]^	488.85	>100/HPF	4+	116 IU/mL	PE/ Blood purification	MHD
15 (this case)	374	797/HPF	2.45 g/24 h	77.107 RU/mL	Pulse dose of intravenous methylprednisolone/rituximab (RTX)/PE/ blood purification/IVIG/sequential steroid combined with tacrolimus (TAC)	Creatinine rechecked was 151.7 μmol/L after 28 wks with erythrocyturia and protein uria improved significantly.

∗ GBM = anti-glomerular basement membrane.

† IgA = immunoglobulin A.

‡ Creatinine = 57–111 μmol/L, Erythrocyturia: -/0–2.4 cells/HPF/0–17 cells/μL, Proteinuria: -/28–141 mg/24 h, Anti-GBM antibody: -/<20 RU/mL.

§ PE = plasma exchange.

|| MHD = maintenance dialysis.

¶ HPF = high power field.

∗∗ MMF = mycophenolate mofetil.

†† IVIG = intravenous immunoglobulin.

The primary therapeutic aim is to render the anti-GBM antibody titer negative as quickly as possible combining plasmapheresis and immunosuppression.^[4]^ Except for patients with double positive for antineutrophil cytoplasm antibody and anti-GBM antibody, relapses are uncommon in patients with anti-GBM disease, maintenance immunosuppressive therapy is unnecessary. Plasmapheresis is recommended in all patients with anti-GBM disease except those who are dialysis dependent at presentation, have 100% glomerular crescents, and do not have pulmonary hemorrhage.^[5,6]^ Glucocorticoids combined with cyclophosphamide is widely used. The addition of anti-CD20 rituximab monoclonal antibody therapy (375 mg/m^2^/wk for 4 wks) has been proposed for patients with severe and/or refractory anti-GBM disease.^[28]^ However, in Heitz et al's report,^[29]^ patients treated with rituximab as a first-line agent for anti-GBM disease showed more significant improvement in pulmonary symptoms, while the renal prognosis was not significantly improved. Similarly, the use of mycophenolate mofetil and cyclosporine has also been reported in individual cases or small series.^[30]^ Compared with the classic cases, even less experience has been reported in the treatment of anti-glomerular basement membrane disease accompanied with IgA nephropathy. There have only been 15 cases^[7–20]^ (including this case) which were reported in the literature, and only 5^[10,12,16,17]^ (33.33%) of them showed significant improvement in renal function after treatment. The success of the initial treatment in this patient was attributed to early pathologic diagnosis and PE. In addition, the innovative use of rituximab in the initial treatment of anti-glomerular basement membrane disease accompanied with IgA nephropathy in this case also enhanced the immunosuppressive effect, which made the pathogenic autoantibodies quickly turn negative, blocking further inflammatory damage, and providing the possibility for the repair of the glomerulus. Our treatment experience also suggests that the treatment of low-dose hormone combined with tacrolimus after PE and intensive immunosuppression is effective, which can not only maintain the negative of pathogenic antibodies for a long time, but also have an effect on the recovery of renal function and even the control of hematuria and proteinuria.

Severe infections were frequent during the early phase of the disease and were associated with substantial morbidity and a reduced 3-year survival rate. The lungs, the urinary tract, and catheters were the main sites of infection.^[21]^ PCP is a severe opportunistic pulmonary infection of pneumocystis jiroveci in immunocompromised patients, which mortality is as high as 29—only 50%.^[22,23]^ In recent years, with the increase of various types of immunodeficiency population, the incidence of PCP shows an increasing trend. In this case, on the basis of acute renal failure and hypoproteinemia, the patient successively received pulse dose intravenous methylprednisolone, immunosuppression with rituximab and plasma exchange therapy, resulting in severe immune suppression and a high risk of secondary pneumocystis opportunistic infection.^[31]^ It has been found that compared with PCP caused by human immunodeficiency virus infection, the secondary PCP of patients with nonacquired immune deficiency syndrome preferred appeared more acute course (5–6 days), 20% of them are less than 3 days.^[32]^ Meanwhile, in our clinical experience, PCP is often characterized by a disproportionate severity of symptoms and signs. In our case, the blood oxygen saturation rapidly decreased and advanced to respiratory failure within 1 day, but there was no obvious fever, coughing, and expectoration. The chest CT showed obvious signs of infection within 3 days, which were consistent with PCP in terms of the rate of disease progression and the clinical manifestations. Pneumocystis include pneumocystis yeii (PJ), pneumocystis carinii, pneumocystis davidii, and other different species of pneumocystis. Among them, PJ is mainly parasitic in the human body and is the most common pathogenic bacteria of PCP. Therefore, the detection of PJ in respiratory tract specimens is judged as the gold standard for the diagnosis of PCP, among which bronchoalveolar lavage fluid has the highest positive rate by microscopy.^[33,34]^ Associated with respiratory failure, especially in patients undergoing mechanical ventilation, the mortality of acute renal failure increased greatly.^[35,36]^ Because of the severe breathing difficulties, and obviously hypoxemia, after weighing the pros and cons, we tentatively used meropenem, linezolid, compound sulfamethoxazole, and caspofungin as a combination of potent anti-infective therapy before etiology results obtained, and rapid remission was achieved within 1 day. In addition, subsequent metagenomics second-generation sequencing also confirmed the diagnosis of pneumocystis pneumonia, so we gradually withdrew the antibacterial drugs. After 14 days of treatment with caspofungin and compound sulfamethoxazole, the chest CT re-examination showed obvious absorption of infection. The patient needed long-term oral immunosuppressive agents after discharge, trimethoprim-sulfamethoxazole (TMP-SMX) is the preferred drug for PCP prophylaxis. However, it has been reported^[37]^ that creatinine reversible increase was observed in 35% patients receiving TMP-SMZ prophylaxis after kidney transplantation, so we only extended the use of TMP-SMZ as prophylaxis for 14 days. In this case, secondary infection was one of the “foreseeable” complications due to the intensive immunosuppressive therapy. The mortality of PCP is very high, and infection significantly increases the risk of renal function deterioration. Therefore, rapid pathogen identification and medication are of great importance, which is one of the key reasons for the successful treatment of this critical case. The infection process of this patient also suggests that, for susceptible patients with severely suppressed immune function, the climate and environment are also key factors affecting the patient's condition. Medical staff should not only have a deep understanding of the condition, but also need full control of the timing and necessity of out-going examination, traumatic diagnosis, and special treatment. In addition, mNGS technology has been gradually applied to the diagnosis of infectious diseases in recent years.^[24]^ Although its clinical practice data still need to be further accumulated, the technology has shown a broad application prospect, but the interpretation of mNGS results still needs to be fully combined with clinical practice.

In conclusion, anti-GBM disease complicated with IgA nephropathy might show symptoms of disease severity and rapid development. Early pathologic diagnosis and PE are the key to the successful treatment of crescent glomerulonephritis, rituximab in the initial treatment also enhances the immunosuppressive effect, which makes the pathogenic autoantibodies quickly turn negative. Low-dose hormone combined with tacrolimus can be used as sequential therapy after PE and intensive immunosuppression. Secondary infection is one of the “foreseeable” complications in this kind of patients due to the intensive immunosuppressive therapy. For patients with acute progression to respiratory failure with severe pulmonary imaging and mild signs, PCP should be considered. mNGS might increase the detection rate of the pathogen, and combined +therapy of caspofungin and compound sulfamethoxazole is safe and effective.

## Author contributions

**Conceptualization:** Dingwei Yang.

**Data curation:** Xiaoxiao Zhang, Xue Li.

**Formal analysis:** Manyu Zhang.

**Project administration:** Manyu Zhang, Weixiu Wang.

**Validation:** Dingwei Yang.

**Writing – original draft:** Manyu Zhang, Fuhao Zhao.

**Writing – review & editing:** Dingwei Yang, Weixiu Wang.
